# Developing an mHealth App for Empowering Cancer Survivors With Disabilities: Co-design Study

**DOI:** 10.2196/37706

**Published:** 2022-07-26

**Authors:** Rachel F Adler, Paulina Morales, Jocelyn Sotelo, Susan Magasi

**Affiliations:** 1 Department of Computer Science Northeastern Illinois University Chicago, IL United States; 2 Department of Occupational Therapy University of Illinois Chicago Chicago, IL United States; 3 Department of Disability and Human Development University of Illinois Chicago Chicago, IL United States

**Keywords:** user-centered design, co-design, mobile health, mHealth, cancer survivors, disabilities

## Abstract

**Background:**

The transition from active treatment to long-term cancer survivorship leaves the needs of many cancer survivors unaddressed as they struggle with physical, cognitive, psychological, and social consequences of cancer and its treatment. The lack of guidance after treatment has forced cancer survivors to manage long-term effects on their own, which has an impact on their overall health, quality of life, and social participation. Mobile health (mHealth) interventions can be used to promote self-management and evidence-informed education.

**Objective:**

This study aims to design an mHealth app for cancer survivors with disabilities that will offer interventions to improve their quality of life and increase their self-efficacy to manage cancer as a chronic condition.

**Methods:**

We organized 3 co-design workshops with cancer survivors (n=5). These workshops included persona development based on data from 25 interviews with cancer survivors with disabilities; prototype ideation, where we sketched ideas for the prototype; and prototype development, where participants critiqued, and suggested improvements for, the wireframes.

**Results:**

These workshops helped us to define the challenges that cancer survivors with disabilities face as well as important considerations when designing an mHealth app for cancer survivors with disabilities, such as the need for including flexibility, engagement, socialization, and a minimalistic design. We also outline guidelines for other researchers to follow when planning their own co-design workshops, which include allowing more time for discussion among participants, having small participant groups, keeping workshops engaging and inclusive, and letting participants dream big.

**Conclusions:**

Using a co-design process aided us in developing a prototype of an mHealth app for cancer survivors with disabilities as well as a list of guidelines that other researchers can use to develop their own co-design workshops and design their app. Furthermore, working together with cancer survivors ensured that the design team had a deeper sense of empathy toward the target users and kept the focus on our ultimate goal: creating something that cancer survivors would want to use and benefit from. Future work will include usability testing of a high-fidelity prototype based on the results of these workshops.

## Introduction

### Background

There are an estimated 16.9 million cancer survivors in the United States, and the number is projected to increase to 22.2 million by 2030 [1]. There is tremendous variability in cancer incidence and survivorship globally because of variation in the prevalence of risk factors as well as access to high-quality preventive, screening, and treatment options [2]. Approximately 40% of cancer survivors experience long-term physical, cognitive, and psychological effects of cancer and its treatment [3,4]. Common long-term effects include pain, fatigue, cognitive effects (eg, cognitive dysfunction or forgetfulness), and psychosocial distress symptoms such as anxiety and depression that can in turn lead to activity limitations and participation restrictions. Long-term effects can negatively affect social participation and health-related quality of life [5]; yet, cancer survivors report that these issues are inadequately addressed within the cancer care system, leaving patients to figure out the impact and long-term management of cancer-related impairments on their own [6]. It is within the purview of the interdisciplinary field of cancer rehabilitation to address the “physical, psychological and cognitive impairments in an effort to maintain or restore function, reduce symptom burden, maximize independence and improve quality of life” [7] of patients with cancer, including those in long-term survivorship. An evidence-informed approach to cancer rehabilitation and survivorship support is the use of self-management interventions that help people learn to deal with the medical, social, and emotional impacts of cancer and its treatment. An emerging body of literature demonstrates that self-management interventions have a positive impact on cancer survivors’ physical and psychological well-being and quality of life [8-10].

It should be noted that interdisciplinary cancer rehabilitation and survivorship services are significantly underused by cancer survivors [11]. Estimates suggest that <10% of the people with cancer-related impairments receive services [12,13]. The reason for this underuse is multifactorial, and in-person access to cancer rehabilitation and survivorship services has been identified as a contributing factor; for example, in the United States, there has been a decentralization of cancer care to favor high-quality cancer care in the community rather than in specialty hospitals. Diffusion of rehabilitation services in these community settings has been limited [14]. Indeed, cancer survivorship and rehabilitation programs are clustered in specialty care centers where fewer than half of the people with cancer receive treatment [15]. Access to rehabilitation services may be further exacerbated for cancer survivors with known disabilities because physical, cognitive, and emotional impairments can make care coordination, scheduling, and travel to and from appointments particularly onerous [7]. Furthermore, evidence suggests that oncologists receive limited education about cancer rehabilitation as well as its benefits and indications [16]. This can make service providers unlikely to initiate referrals for rehabilitation services. In addition, most National Cancer Institute–designated cancer centers do not provide information about cancer rehabilitation services on their websites, further restricting access and awareness of these potentially beneficial interventions [17]. This leads to a gap between the people who need support and those who receive it.

Mobile health (mHealth) interventions have been identified as a way to close the gap between the people who need rehabilitation services and those who are able to access them. mHealth interventions have a potentially democratizing impact on access to care because people are able to take in their own hands the tools to monitor and manage their health. mHealth interventions can provide people with tools to promote self-management, symptom monitoring, and evidence-informed education [18], as well as opportunities for peer support and information sharing [19]. Although a variety of mHealth symptom management apps have been developed for cancer survivors, the apps tend to focus on monitoring and managing individual symptoms such as pain and fatigue [10]. As a result, existing mHealth self-management interventions often fail to address the knowledge and skills that cancer survivors with known disabilities need to achieve their goals of creating a meaningful life in spite of the aftereffects of cancer and its treatment [5]; for example, our qualitative research with cancer survivors indicated that many people were uncertain of how and when to communicate about the impact that the aftereffects of cancer had on their abilities to fulfill their social roles and responsibilities [20]. These findings point to an unmet need for strategies to help survivors articulate the impact of cancer on their daily lives and to self-advocate for support and accommodations.

### Objectives

To address the unmet needs of cancer survivors who can benefit from ongoing support, we are developing a self-management intervention for cancer survivors with known disabilities called WeCanManage. WeCanManage is conceptualized as a psychoeducational intervention theoretically grounded in Individual and Family Self-Management Theory [21]. The active ingredients of the intervention include instruction and structured practice in (1) the problem-solving–based self-management process of goal setting and action planning supplemented with evidence-informed strategies such as energy conservation and environmental modifications, (2) mindfulness practices [22], and (3) self-advocacy skills. These complementary approaches empower users to build their self-efficacy, the presumed mechanism of change [23], in medical management, role management, and emotional management of cancer as a chronic condition [24]. Consistent with emerging best practices for remote learning, content will be delivered through mobile microlearning modules [25] for a maximum of 10 minutes per day over a 4-week period. Engagement activities will be embedded across the intervention to promote deeper learning and integration into daily life and routines [26,27]. We plan to deliver WeCanManage as an mHealth app, because internet-based self-management interventions provide users with a practical, flexible, and cost-effective alternative to face-to-face interventions [28-32].

For mHealth and rehabilitation tools to be acceptable, accessible, and responsive to the needs of the intended users, Jones et al [33] highlight the importance of proactive engagement of stakeholders, including members of the disability community. However, patient input is rarely included in the development of self-management interventions [8]. To ensure that the WeCanManage platform and design meets the needs and preferences of our target end users, we engaged a cohort of cancer survivors in the design process.

Whereas user-centered design is the process of focusing on users and their needs throughout the stages of the design process [34], co-design, or participatory design, takes this process one step further where designers and users collaborate during the design process [35]. Co-design can be valuable when developing health-related apps [36,37] and can incorporate engaging techniques such as scenarios or storytelling approaches [38], developing or discussing personas [39-41], reporting on likes or dislikes regarding apps [42], voting on features and solutions [43], sketching out prototypes [38,44,45], redesigning or critiquing prototypes [36,40,46], and answering questionnaires [41,45]. Supplies can be minimal, such as paper, Post-it notes, and posters [45,47,48].

In our work, we primarily use 2 common co-design techniques: persona development and prototyping. Personas are fictional representations of users that help designers to understand and empathize with users [49,50] by challenging them to think “beyond their personal experiences” [39]. Each persona can have a set of characteristics, such as their gender, age, profession, goals, personal history, health issues, technological skills, and hobbies; often, a photograph is included [50]. Personas can include disabilities, which can raise awareness of accessibility and ensure that a design can be used by all [51]. Cocreating personas with people with disabilities can provide even more insight into their experiences [52]. Despite the fact that personas are not real people, they are often developed based on analysis of common themes discovered during user interviews [51,53,54]. Co-design workshops can be effective alongside user interviews [55], particularly because they can be helpful for persona creation. Another common activity during co-design workshops is prototyping. These are often wireframes (low-fidelity sketches) of a potential design that can help lead to higher-fidelity prototypes that are functional and closer to the finished product [56].

To build our intervention and app design, we recruited cancer survivors with disabilities to work with us toward creating a persona (workshop 1) and a prototype of an mHealth app that would empower the community of survivors to self-manage the lifelong effects of their cancer treatment (workshops 2 and 3).

Our research questions (RQs) are as follows:

- RQ1: What are the important design features for an mHealth platform for cancer survivors with disabilities?
- RQ2: What is needed to create an effective co-design environment for this target group?

## Methods

### Recruitment

To design an mHealth app for cancer survivors with disabilities, we recruited a diverse group of cancer survivors with known disabilities (n=5), whom we call *survivor scientists*, using a citizen scientist approach, to collaborate with our interdisciplinary research and development team [57]. Our inclusion criteria included participants self-identifying as a cancer survivor living with long-term physical, cognitive, or social effects of cancer and its treatment and that they had an established relationship with a faculty member or an institutional or organizational partner. The survivor scientists had experienced breast cancer, head and neck cancer, sarcoma, brain cancer, and leukemia, as well as a range of long-term effects of cancer (including cognitive changes, visual impairments, communication challenges, and decreased functional mobility and fine motor control). All (5/5, 100%) of the survivor scientists were cancer-free and at least 5 years after diagnosis and completion of primary treatment. Several (3/5, 60%) of the survivor scientists are also active in cancer and disability advocacy organizations. The survivor scientists have a variety of professional backgrounds, including social work, graphic design, research support, and rehabilitation medicine with certification in cancer rehabilitation.

Together with the 5 survivor scientists (n=2, 40% men and n=3, 60% women), our team led 3 co-design workshops from July 2021 to October 2021. The first 2 workshops were conducted 2 weeks apart in July, and the third was conducted approximately 3 months later in October to provide enough time for our team to implement wireframes based on feedback from the previous 2 workshops. It should be noted that after the second workshop, we also provided the survivor scientists with the opportunity to continue to work with the research team in smaller groups and assigned them tasks related to their own interests, such as helping with content relating to cancer and disabilities. This work continued after the third workshop as well.

The participants received monetary compensation for their involvement in the co-design process. These workshops included (1) persona development, (2) prototype ideation, and (3) prototype development (wireframes). We developed a semistructured guide for each workshop based on the guide provided in Bradway et al [58]. Refer to Multimedia Appendices 1-3 for our guides for all 3 workshops. The workshops consisted of the design team (8 researchers, faculty, and students from the Departments of Computer Science and Occupational Therapy and Disability Studies from 2 universities) working in combination with our 5 survivor scientists. It should be noted that our last workshop had fewer participants because, of the 5 survivor scientists, 1 (20%) could not attend; in addition, of the 8 members of the design team, 1 (13%) researcher and 2 (25%) undergraduate students who had completed their research experience did not attend; therefore, the total number of participants went from 13 (n=8, 62%, design team members and n=5, 38%, survivor scientists) to 9 (n=5, 56%, design team members and n=4, 44%, survivor scientists). Because of COVID-19–related restrictions, all 3 workshops were held over Zoom, a videoconferencing platform. The workshops were video recorded. The first workshop lasted for 2 hours, and the remaining 2 workshops lasted for 2.5 hours each. This duration is consistent with previous research [40], and we chose an amount of time that would be long enough to accomplish our goals and still keep participants engaged but not too long, particularly because the workshops were conducted on the web, after work, and with participants who had long-term disabilities as a result of cancer.

Within a week after each workshop, the design team met and summarized the main findings from the workshop. These notes were used to inform the development of the workshops that followed; for example, based on the challenges discovered in workshop 1 and the prototype features that the participants mentioned wanting in workshop 2, we developed the low-fidelity prototype that was then shared with the participants in workshop 3 for their feedback.

Although our goal was to create an mHealth app for cancer survivors with disabilities that (1) normalized their experiences as a survivor, (2) taught problem-solving self-management skills, (3) introduced mindfulness-based practices, and (4) addressed self-advocacy skills and disability and survivor rights, the goal of the co-design workshops was to ensure that we would be designing these modules in a way that was usable and engaging to cancer survivors with disabilities.

### Ethics Approval

We obtained institutional review board approval from the participating universities in the larger project (University of Illinois Chicago #2020-1067, Northeastern Illinois University #79, and Northwestern University #NUUIC21CC03). The survivor scientists functioned as members of the design team, and no personally identifying data were gathered about them. Ground rules for participation to ensure respect and confidentiality throughout the process were established and agreed to by all participants. All cancer survivors who participated in formative qualitative interviews provided written informed consent before data collection in compliance with approved institutional review board protocols at the collaborating institutions. More details on the interview process and findings from this phase of the study have been reported elsewhere [20].

## Results

### Workshop 1: Persona Development Methodology

**Figure 1:**
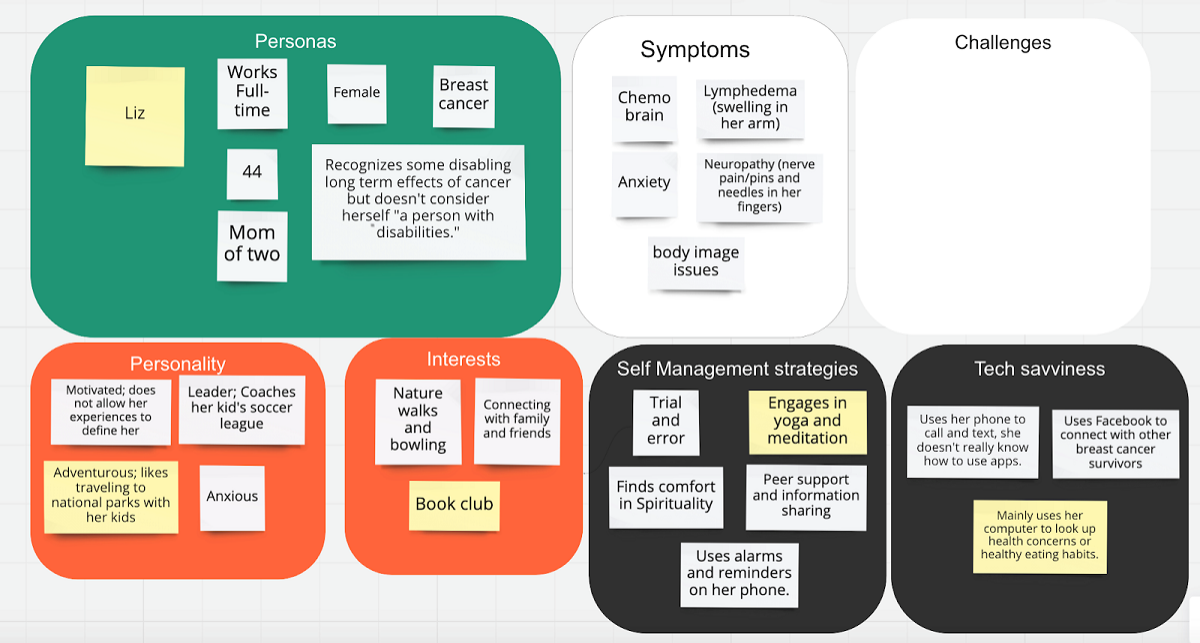
Sample persona before the workshop.

The aim of our first workshop was to complete the design of personas to help the design team understand the needs and challenges of cancer survivors with disabilities and build empathy through persona development. The survivor scientists (n=5) attended our first persona development workshop and worked alongside our team of researchers. In advance of this workshop, the research team created 2 personas of cancer survivors with disabilities, using Miro, a visual collaboration platform. These personas were developed based on preliminary analysis of 25 qualitative interviews of breast cancer, sarcoma, and head and neck cancer survivors to foster empathy for the struggles of survivors living with long-term disabilities. Specifically, demographic and clinical data were extracted from the data corpus (demographic surveys, interview transcripts, and field notes) to ground the preliminary personas in the lived experience of survivors of breast cancer, head and neck cancer, and sarcoma. Conceptually, we structured our personas in accordance with the World Health Organization’s International Classification of Functioning, Disability, and Health (ICF) framework [59]. The ICF is an internationally recognized biopsychosocial framework that recognizes the dynamic interaction between impairments in body structures and function, activity limitations and participation restrictions, and environmental factors in people’s lived experiences of disability. Extracted data included age, gender, race and ethnicity, cancer type, primary symptoms, and impact on social roles and participation. Although our work was informed by the literature [50,51] and the ICF, the categories chosen were based on common themes from our analysis that would make the personas more relatable. We input the data into a persona template provided by Miro to create 2 distinct personas. We decided not to include an image to avoid participants accepting what we provided as a given, but we wanted them to improve upon and complete the profiles. Figure 1 shows a sample of one of the personas developed for the workshop. The challenges section was intentionally left blank for the survivor scientists to fill in that category themselves.

We used 2 breakout rooms to discuss, modify, and complete each persona. Using a Zoom poll, the survivor scientists ranked the most relevant challenges that a cancer survivor was likely to face. Finally, we discussed the ranking results with the survivor scientists.

### Workshop 1: Persona Development Results

**Figure 2:**
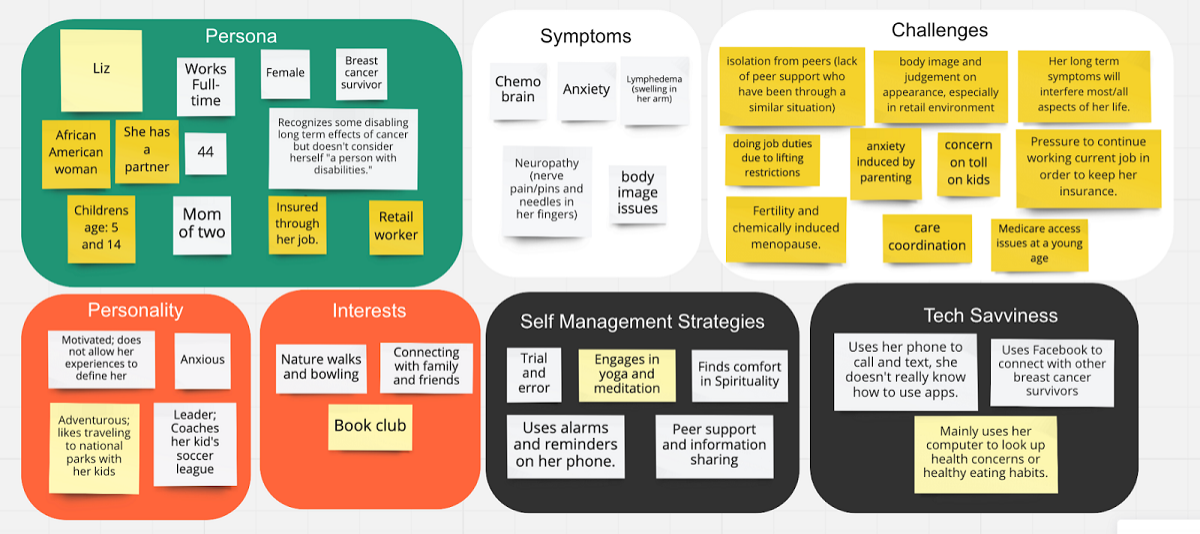
Sample persona after the workshop.

During these breakout sessions, the survivor scientists critiqued the personas and described the needs and challenges; for example, in breakout room 1, the survivor scientists modified the persona for Liz, a breast cancer survivor, by providing more details making her even more relatable (Figure 2). Liz is now an African American woman, with a partner, and children aged 5 and 14 years. She works in retail, which requires prolonged standing and heavy lifting and has limited insurance tied to her job. The survivors discussed challenges, some of which include financial pressures, long-term symptoms interfering in her life, isolation because of lack of peer support, concern regarding the emotional toll on her children and relationship with her partner, and body image concerns. The survivor scientists felt that these details helped to encapsulate the complexity of living with the long-term effects of breast cancer while juggling multiple roles and responsibilities.

In breakout room 2, the survivor scientists discussed Solomon, a man aged 56 years, who is divorced and is on the fence regarding looking for a romantic partner because of body image issues as a result of head and neck cancer. His interests originally included karaoke and barbecues with his family most weekends. However, the survivor scientists discussed how he may have once enjoyed karaoke and eating with his friends before cancer, but now singing would be difficult, and eating with friends would cause anxiety because of his slower eating speed. The results of both breakout sessions showed that the survivor scientists deemed it important to acknowledge the high levels of anxiety caused by role loss, fear of recurrence, financial toxicity, and the strain that cancer continues to impose on relationships with family and friends. In addition, the survivor scientists wanted to highlight the challenges that cancer survivors encounter when re-establishing leisure roles and maintaining their employment because of physical and cognitive limitations, as well as the impact that role loss has on a person’s identity and sense of self. Use of compensatory strategies and adaptation were also discussed.

**Figure 3:**
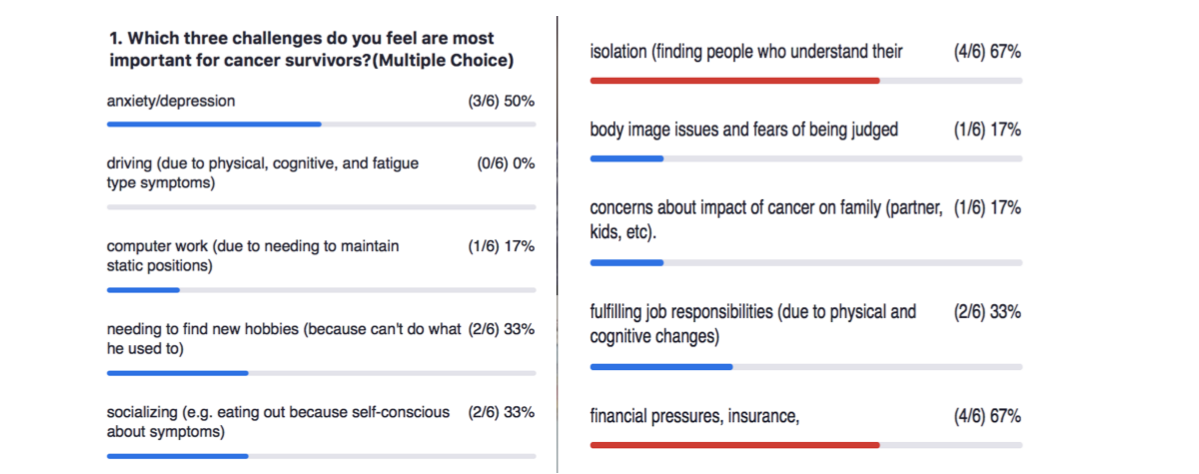
Ranking the results of the persona workshop.

After the breakout sessions, participants reconvened in the larger group and shared their enhanced personas. Using the challenges identified in both breakout sessions, we had participants select the 3 challenges that they felt were most important using Zoom’s polling feature. The results, presented in Figure 3, reveal that isolation, financial pressures, and anxiety or depression were the biggest challenges. These were closely followed by participation in work and leisure, as well as social roles. It should be noted that 6 participants completed the Zoom poll because, of the 8 members of the design team, 1 (13%), a researcher, is also a breast cancer survivor and provided feedback alongside the survivor scientists based on her own experiences as a survivor.

### Workshop 2: Prototype Ideation Methodology

The aim for the second workshop was to determine what features cancer survivors with long-term disabilities like in current apps. We first asked the survivor scientists to list (in the Zoom chat) the apps they like in general as well as the apps they dislike to encourage them to think about design. We then directed them to a shared Google Sheets document where they each had a column with their names to list the features they liked in apps and the features they did not like. We generated a word cloud using a free word cloud generator [60] with the results. Next, we divided participants into 2 breakout rooms (intentionally placing them with different people than at the previous workshop as well as keeping in mind diversity in terms of disability and gender). We showed each group different potential content and asked them how they would deliver this content so that it would be inclusive and engaging to cancer survivors. Using the Miro board, we also asked them to provide us with sketch ideas for how they envisioned the app. Although there were design limitations in terms of technical capabilities and budget on the development side, we told the survivor scientists to think in terms of an open universe where anything is possible because we did not want to limit their thinking. Once merged back into the larger group, each group presented their work to the other group. Using features that both groups felt were important in the design of the app, we had the participants rank the features that were most important to them using Slido, a live polling platform (Cisco Systems, Inc).

### Workshop 2: Prototype Ideation Results

**Figure 4:**
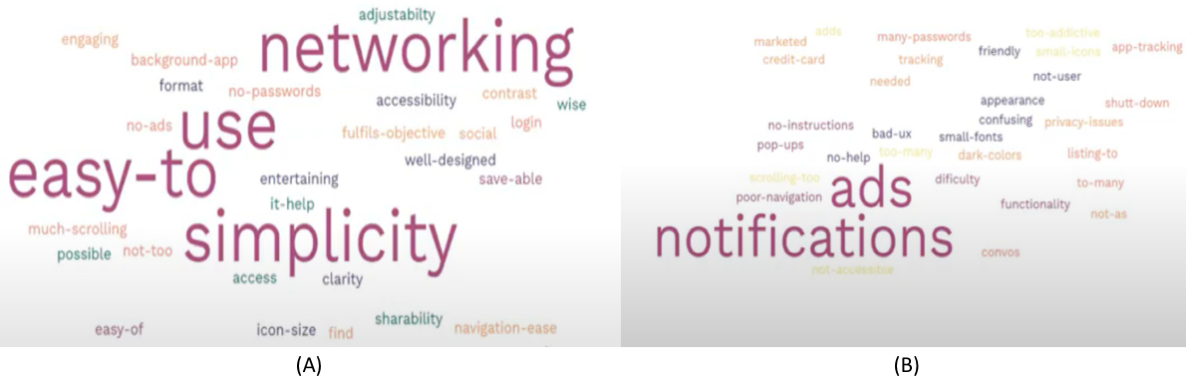
(A) Participants’ likes in apps. (B) Participants’ dislikes in apps.

After the participants had listed their likes and dislikes in apps, we generated a word cloud (Figure 4). The features that the survivors liked included networking, ease of use, and simplicity, whereas the features that the survivors disliked included lack of user-friendliness, too many notifications and advertisements, and poor navigation.

**Figure 5:**
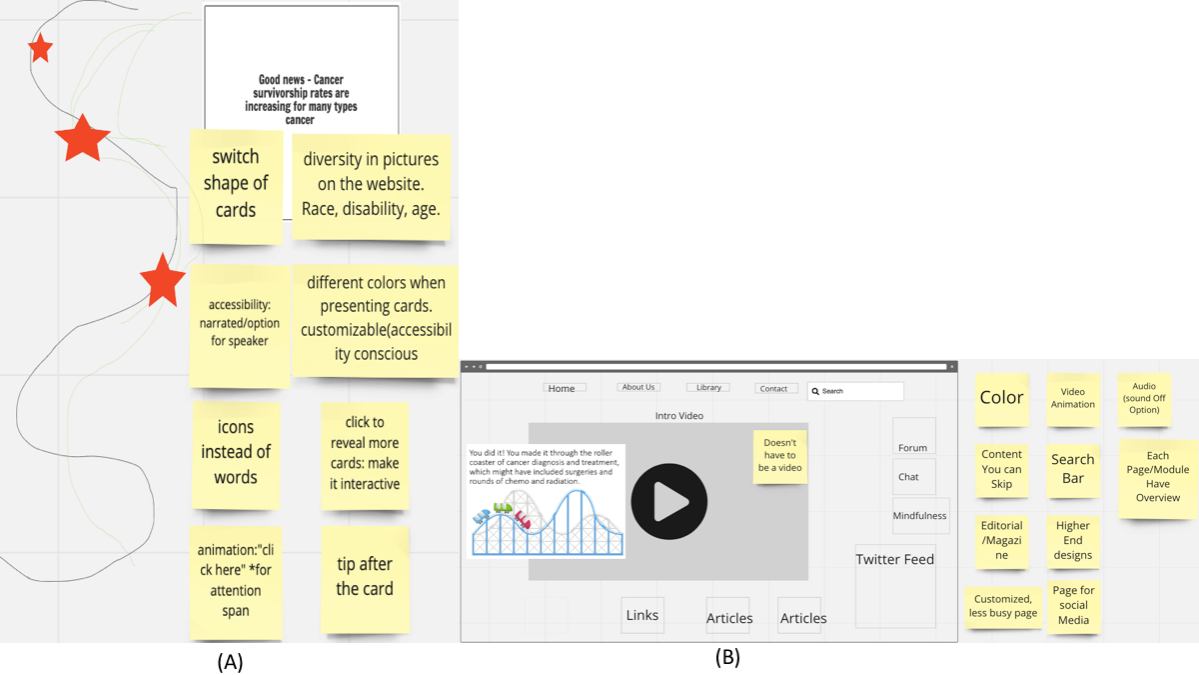
Workshop sketches. (A) Breakout room 1. (B) Breakout room 2.

After we divided the survivors into 2 groups and asked them to think about ideas for sketches (using Miro) that they envisioned for the app, one of the groups asked the workshop leader to sketch a learning pathway to be able to provide ease of navigation and engagement (Figure 5A). The participants also mentioned that the app should include photos of people from different backgrounds in terms of race, disability, and age. In addition, they discussed including the use of animation, which can help those with attention disorders, icons instead of only text for those with cognitive disabilities, and customizable color choices and simpler layouts for those with visual impairments. In the second breakout room, of the 5 survivor scientists, 1 (20%) created a paper mockup that she felt would look engaging, and based on her drawing which she held up to the camera, along with feedback from the rest of the breakout room participants, workshop leaders sketched it using the Miro board (Figure 5B). The survivor scientists felt that the content should be engaging, with videos as well as content that was easy to access. Furthermore, they discussed that apps for people with disabilities tend to be poorly designed and “ugly” and that good design would alienate no one. They were very happy to be part of the process of designing the app.

**Figure 6:**
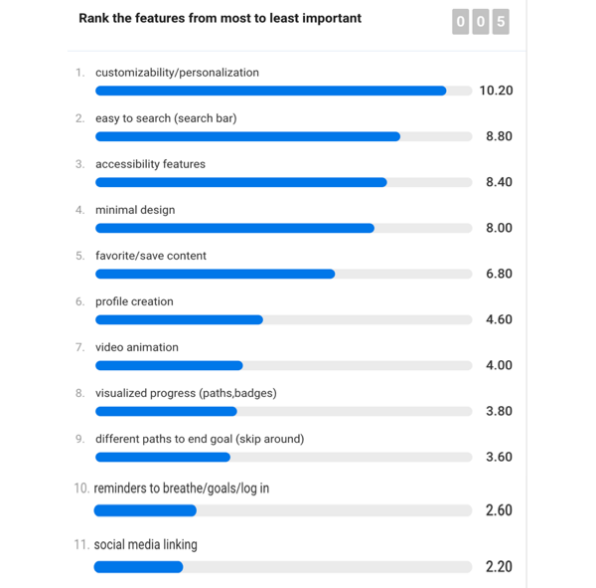
Features ranked from most important to least important.

After gathering all the information that the survivors added to the sticky notes in Miro, the word cloud, and the sketches, we asked the survivors to rank the features from most important to least important (Figure 6). The top 3 features that were most important to the survivor scientists were customizability and personalization, ease of navigation and searchable content, and accessibility. This was followed by minimal design.

### Workshop 3: Prototype Development Methodology

**Figure 7:**
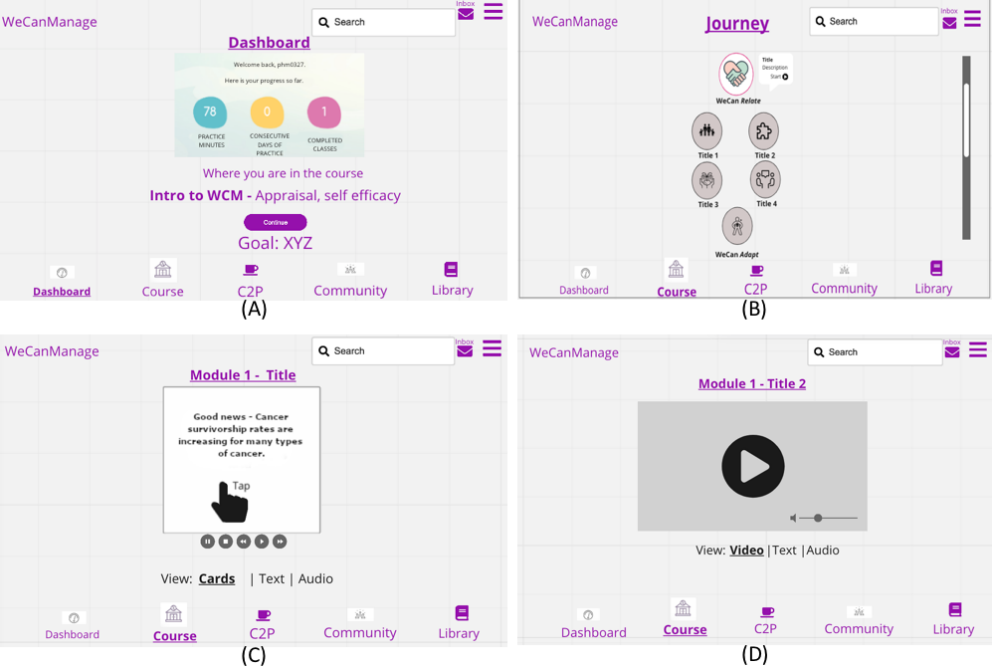
Wireframes. (A) Dashboard page. (B) Course navigation. (C) Cards module. (D) Video module. C2P: Connect to Peers; WCM: WeCanManage.

In our third workshop, we wanted to find out what the participants thought of the low-fidelity prototype that was created to address the challenges raised in workshop 1 (eg, isolation) and the features they mentioned wanting in workshop 2 (eg, customization). We also wanted to learn what improvements could be made. We divided the researchers and survivor scientists into 2 breakout rooms to critique the wireframes. After coming together in the larger Zoom room, both groups presented their thoughts on the design. We also led a conversation on whether to include a community forum or a feed-like aspect in the app. As shown in Figure 7A, the first screen presented to the survivor scientists was the Dashboard. To meet the survivor scientists’ needs for personalization, we incorporated a section for a personalized goal that the user would type in and be working toward achieving. This would be visible on the landing page after logging in. We also matched the pathway that the survivors requested from the previous sketch (Figure 5A) in the Course tab (Figure 7B). Connect to Peers (C2P; the C2P tab) was provided as a space for users to build their network and connect with other cancer survivors through direct messaging, with the Community section being a place where users can share their experience and support one another through forums. The Library section would contain helpful resources. When logging into a course, the users will be offered a series of microlessons subdivided into 4 content modules: *WeCanRelate* seeks to normalize and validate their experiences as survivors, *WeCanAdapt* focuses on problem-solving–based self-management skill building to promote self-efficacy, *WeCanBreathe* introduces mindfulness-based practices, and *WeCanSpeakUp* addresses self-advocacy skills as well as disability and survivor rights. Users will also be given the opportunity to select the mode of presentation they prefer, which includes formatting the participative cards (or video) into text or audio (Figures 7C and Figures 7D). This will allow users to comfortably access the content in the mode that best supports their learning style and access needs. We also provide knowledge checks throughout each course module.

### Workshop 3: Prototype Development Results

**Figure 8:**
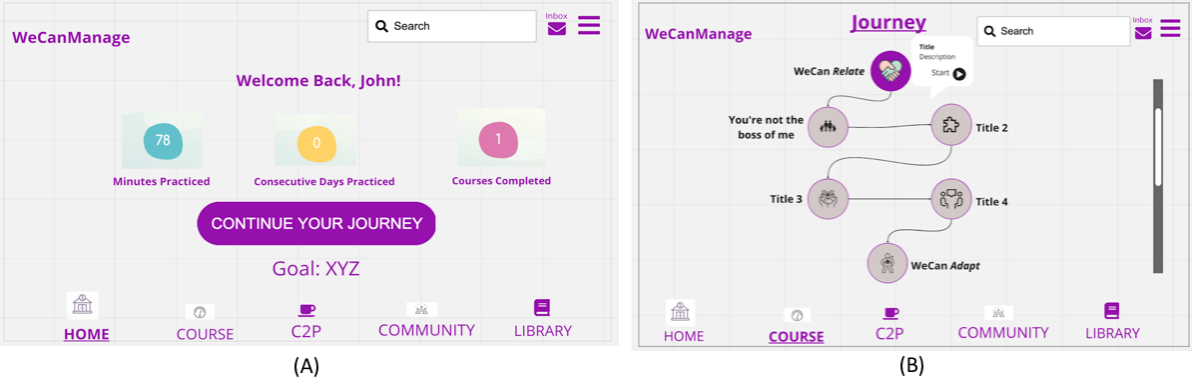
(A) Updated home screen. (B) Updated Journey page. C2P: Connect to Peers.

On the basis of the results of the workshop, the survivor scientists liked the wireframes but had concerns with the main Dashboard screen having too much information and the Journey screen having too much white space, and they did not like the terminology used, such as the words dashboard, course, and quiz. Figures 8A and Figures 8B show sketches of the home screen and Journey page, respectively, revised after discussions with the survivor scientists.

Although we had a larger group discussion on whether to include community forums or a feed-like design (as available on many social networking sites), the participants did not have a strong preference for one format over the other. The survivor scientists liked that there was a way to contact peers on the site either directly or through the forum or feed option.

## Discussion

### Principal Findings

We formulated our RQs to discover what design features are important for an mHealth platform for cancer survivors with disabilities (RQ1) and what would be needed to create an effective co-design environment for this target group (RQ2). The 3 workshops provided us with a strong foundation to move forward with well-defined goals for designing an mHealth app for cancer survivors with disabilities. Our results indicate that participants wanted an mHealth app that encompasses (1) flexibility, (2) engagement, (3) socialization, and (4) a minimalistic design.

Our first finding is that participants wanted *flexibility* in the app. The top-ranked feature that the survivor scientists wanted in workshop 2 was customization and personalization. Participants wanted an app that can be easily customized to their preferences and their access needs. They also wanted accessibility features and an app that was easily searchable. Therefore, in our design for workshop 3, to suit the content to the learning needs of the individual we provided the option to view content (whether video or cards) in an audio-only or text-only format, providing flexibility to the user.

Furthermore*,* the survivor scientists, in workshop 2, reported liking features that were participative. Users are more receptive to mHealth apps when they are *engaging* [61]. The survivor scientists recommended the use of color, video, and interaction. They also liked the idea of a learning pathway for the course content. We incorporated this feedback in the prototype shown to the participants in workshop 3. As noted in the study by Jessen et al [40], participants liked receiving recognition from an mHealth app for finishing tasks and setting up their own goals and subgoals. Similarly, we found that participants liked being able to input their goals and view them on the main screen. In addition, we discovered that participants enjoyed ways to determine their progress through quiz-like activities and a journey section that kept track of their course pathway.

The participants reported in workshop 1 that one of the biggest challenges was isolation. Although engaging features can be beneficial in an mHealth app, communicating with others going through similar experiences has been found to be a key feature in supporting mHealth apps [40]. Therefore, we found that survivor scientists wanted *socialization* as well. In our design, we showed participants in workshop 3 the different ways in which users could interact with each other. Participants liked having a way to connect directly with peers going through similar experiences (the C2P section) and benefit from being able to ask questions or post comments to the community of cancer survivors (the Community section). This enhanced their socialization experience.

Although including goals, progress, and opportunities for socialization are effective techniques, similar to other studies we found that the app should be designed using a *minimalistic design* approach [62]. In workshop 2, our participants reported preferring minimal design. This concept was supported again in workshop 3, when participants reported not liking when there was too much content on the screen. They wanted a clean and simple interface that would engage them and allow them to easily comprehend where to go. This was most noted on the home screen, which they felt was initially too cluttered.

### Comparison With Prior Work

The study was able to extend previous research by addressing RQ1 and providing tips on creating mHealth apps through what we learned in our co-design workshops (flexibility, engagement, socialization, and a minimalistic design). Furthermore, our work extends previous work on planning successful co-design workshops for health-related apps [36,37], particularly for internet-based co-design workshops. Näkki and Antikainen [63] found that using web-based tools can make it easier and cheaper to include users as co-designers. People with disabilities benefit from being able to work in remote settings, which therefore promotes a more inclusive environment [64]. The Zoom-based co-design workshops, despite their limitations, provided flexibility for the survivor scientists in terms of geographical limitations and participants with disabilities being comfortable and able to work out of their own homes. In our work, to address RQ2, we found that successful co-design workshops should be engaging, inclusive, provide more time for participants to speak up, use smaller participant groups, and let participants think big (provide a universe where anything is possible).

Researchers should keep the co-design process as *engaging* as possible while keeping in mind their target users, the technology, and any limitations [40,58]. Although keeping it engaging is true in face-to-face workshops as well, this can be even more pronounced in a web-based environment where “Zoom fatigue” [65] can be prevalent. Engaging techniques can be included in numerous ways. In our case, we incorporated many web-based tools such as Zoom’s breakout rooms, poll and chat features, Slido, word clouds, and Miro.

In addition, to help keep it engaging, a significant portion of time should be allotted *for participants to speak up*. Arsand and Demiris [38] discuss allocating sufficient time for several meetings with users to allow time for their creative ideas. In our first workshop we had many more activities planned that we did not get to complete because we did not allocate enough time for participation of the survivor scientists. We made adjustments by adding the needed time and not finishing everything as planned; for example, we had allocated 20 minutes for the breakout session in workshop 1, which we modified to 30 minutes while in the breakout rooms. We also provided 10 minutes for the survivor scientists to discuss their challenges, but this was not enough; therefore, we continued to let them talk and decided not to complete all the topics that came later in the list included in the workshop guide. Staying flexible allowed us to provide additional time to the participants. Additional time for conversation may be particularly important in a web-based format, which limits some of the informal communication and connections inherent in in-person workshops. Learning from this, we set the time allocated for the next 2 workshops to 2.5 hours (instead of 2 hours) and provided extra time for the breakout rooms and survivor scientist discussion (refer to Multimedia Appendices 2 and 3). Finally, we reduced the amount of time that we presented to make it more engaging and participatory. Providing survivors with additional time and opportunities for feedback and sharing of ideas in the workshops was crucial because many of their ideas helped to shape the design of the app.

For participants to be comfortable speaking up and participating, previous work has shown that breaking out into *smaller working groups* can be helpful [66,67]. Using Zoom breakout rooms in web-based workshops can foster engagement and reduce “Zoom fatigue” [67]. We only included 5 survivor scientists, but, with the addition of the researchers, workshops 1 and 2 had 13 participants each. Although the breakout rooms were very helpful in facilitating the division of the team into smaller groups, after the second workshop we also recruited survivor scientists to work individually with a researcher on content development or design; for example, of the 5 survivor scientists, 1 (20%) came up with the idea of modifying WeCanManage course content into modules whose titles will all begin with WeCan; for example, WeCanRelate. Another survivor scientist offered to work on content development for specific cancer symptom management resources, whereas another will focus on more of the disability aspects. Having survivor scientists self-select into areas based on their own skills and interests enabled them to be even more efficient and feel useful in the co-design process.

Another approach to encourage participants to speak up is through creating an *inclusive environment*. Creating a comfortable and inclusive environment in co-design workshops is one of the lessons found in the study by Bradway et al [58]. Similarly, we wanted the environment to be welcoming to all. Therefore, we began the first workshop with time for rapport building and informal conversation before establishing ground rules aimed at creating an environment of trust and respect. The ground rules included emphasizing that everything discussed in the workshops was confidential, and all opinions were valid. We reminded participants of this at the beginning of the next 2 workshops. Although there may be differing opinions, it is important to show respect for all the different views, particularly because participants can be very passionate about an app that would be of direct benefit to them. Although there are a limited number of inclusive apps [68], ensuring respect for co-designers with their own particular backgrounds and disabilities can lead to the creation of apps that are more inclusive because the co-designers keep in mind their own personal experiences and disabilities in the design process.

In addition to making co-design participants feel comfortable sharing their ideas, it is important to allow them “the latitude to dream big and imagine a best-case scenario with no constraints” [69]. The concept of *dreaming big* and providing a universe where anything is possible can promote creativity and facilitate learning about what users find important in a design [69-72]. To encourage participants to speak up, we did not limit their ideas by mentioning technical or budgetary considerations but encouraged them to dream big. Particularly in a web-based co-design workshop, dreaming big could be even more difficult for participants with limited technical skills. However, we saw how important and useful this was throughout the design process; for example, although the learning pathway sketch (Figure 5A) was how the participants were picturing the design to be, after speaking with our development team we toned it down to a parallel tile-based design (Figure 7B). Later, this was modified after further conversations with the survivor scientists, using a compromise approach that worked for the development team as well as the design team (Figure 8B). Having co-designers who are not limited to the practical aspects of design can help to encourage creative thinking and lead to a more meaningful design.

### Limitations

We encountered limitations in the co-design process because of technical difficulties and pandemic-related hurdles. Because of COVID-19–related restrictions, we conducted all workshops with survivor scientists over Zoom. Although this provided flexibility in terms of location and scheduling, we were limited in that we could not use Post-it notes, posters, and paper during the workshops. We conducted most of our designing of personas and prototypes through Miro. As the technology can be challenging for first-time users, we chose to have 1 person from our design team lead the Miro board activities in each breakout session.

### Conclusions

The results from the co-design workshops provided our research team with a deeper level of empathy for our target users and a better understanding of long-term survivorship challenges and needs for an mHealth app. The collaborative development aided in creating a shared vision of target users among researchers and survivor scientists, while being an engaging co-design experience. Future work will continue to include survivor scientists in the design process as we create a high-fidelity prototype and conduct usability testing, which will be followed by the implementation of the app.

## Appendix

Multimedia Appendix 1 A guide for workshop 1: persona development.

Multimedia Appendix 2 A guide for workshop 2: prototype ideation.

Multimedia Appendix 3 A guide for workshop 3: prototype development.

## Abbreviations

C2P: Connect to Peers
ICF: International Classification of Functioning, Disability, and Health
mHealth: mobile health
RQ: research question
