# Supplementary material for: Developing an mHealth App for Empowering Cancer Survivors With Disabilities: Co-design Study
Source: JMIR Form Res. 2022 Jul 26;6(7):e37706. doi: 10.2196/37706 (PMC9364172; doi:10.2196/37706)
Supplement: Multimedia Appendix 1 [file formative_v6i7e37706_app1.docx]

**A Guide for Workshop #1 Persona Development**

| **Aim *(what)* and Purpose *(why)*** | **Time** | **Activity** | **Practicalities and Instructions** |
| --- | --- | --- | --- |
| Aim: To familiarize participants with activities and each other | 5 minutes | Welcome & Team Introduction | Team Introductions: Name, Title, Institution, What you bring to the team |
|  | 7 minutes | Introductions | Survivor Scientist Introductions - Name, cancer type, how far out you are, one thing that surprised you of being a survivor, fun fact *about themselves* |
|  | 3 minutes | PowerPoint | Agenda |
|  | 3 minutes | Workshop Rules | - Everything in this workshop will remain confidential. - Try to leave camera on - Mute microphone when not talking - Recordings will only be used for research purposes. - Identifiable info when recording/introductions - Everyone’s opinions are valid - Honest feedback is best to improve research - Gentle reminders will be given if we get off track - Any other ground rules that you’d like to establish? |
|  | 10 minutes | Description of project purpose and aim | WeCanManage Introduction  Purpose   - *What we want to know?* - *How we can help?* - *Goals?* |
|  | 6 minutes | Co-Design Process | Introduction to Co-Design   - *What is Co-Design?* - *Why is it beneficial?* |
|  | 10 minutes | Case studies & progress | Introduction to qualitative interview findings so far and individual case studies |
|  | 3 minutes | Personas | *Introduction to Personas Workshop*   - *Introduce workshop based on powerpoint.* |
| **Aim:** Critique personas and build depth  **Purpose:** Identify and understand the needs of cancer survivors with disabilities that our mHealth app can serve | 20 minutes | Personas Breakout Room | **Group Discussion:**  Split into 2 groups  Critique Personas, leave blank post it notes to be filled out, add or remove post-it notes as needed in the persona.  **Building depth (guiding points for facilitator):** Tell them to think about: Personality traits. Impacts of the disability/cancer? Habits? Preparations/Routines? Discuss any further challenges the persona might face.  Challenges Present: How do cancer and its long-term impacts impose challenges on things that Liz/Solomon needs or wants to do (this can be physical, social, emotional, as well as engagement in self-care, productive and leisure roles). |
|  | 10 minutes | Present Persona | ***Come together after discussion, present the persona and continue with ranking the challenges. Are there any volunteers to present our personas?*** |
|  | 10 minutes | Challenges | **Ranking Challenges:** Which are the most pertinent to least pertinent?   - Make a poll *(zoom ranking)* - *Discuss ranking* |
|  | 15 minutes | Needs/Solution | Needs/Solutions/Problem Solving (use post-its in Miro)  What are some needs based on these challenges?  Solutions?  What types of ways for them to problem solve? |
| Aim: Get survivor scientists to start thinking in terms of design.  Purpose: Prime survivors for the next workshop | 10 minutes | Bad & Good Apps | *Put in the Chat:*  *Name Bad Apps in general you have used.*   - *And why you think they are bad*   *Name Good Apps in general you have used.*   - *Why are they effective?*   *Discuss the above bad and good apps.*  *Discuss: Future Good App - doesn’t have to exist yet. What would you like to see in an app in general (if time)* |
| Aim: Close off and thank participants.  Purpose: Show appreciation for their time and better inform future co-design workshops. | 3 minutes | Closing Remarks | Thank you  What is to come?  Next Session: Starting Prototypes |
