# Supplementary material for: Developing an mHealth App for Empowering Cancer Survivors With Disabilities: Co-design Study
Source: JMIR Form Res. 2022 Jul 26;6(7):e37706. doi: 10.2196/37706 (PMC9364172; doi:10.2196/37706)
Supplement: Multimedia Appendix 2 [file formative_v6i7e37706_app2.docx]

**A Guide for Workshop #2 Prototype Ideation**

| **Aim *(what)* and Purpose *(why)*** | **Time** | **Activity** | **Practicalities and Instructions** |
| --- | --- | --- | --- |
| Aim: To familiarize participants with activities and each other | 10 minutes | Introduction | Welcome  -Record session - all opinions valid, speak one at a time, confidential/no identifiable  -Overview of the agenda for the day   - Warm up - Workshop - Discussion - Closing remarks   Brief introduction of the survivor scientists and team, if new participants arrive to meeting   - Name and another fun fact |
| Aim: Get survivor scientists to start thinking in terms of design.  Purpose: Prime survivors for the main portion of the workshop. | 20 minutes | Worst & Best App Warm-up | *In Chat:*   1. *Current Good Apps you like* 2. *Current Bad Apps you dislike*   *In Spreadsheet:*   1. *Type one word answers (or hyphenated) for things you like that keep you motivated to come back to an app?* 2. *Type one word answers (or hyphenated) for things you dislike in some Apps?*   *Discussion:*   - *Recap Show Word cloud of 3 and 4* - *What are Future Good features that don’t currently exist?* |
| Aim: Better understanding of content and engagement.  Purpose: Prime survivor scientists for layout design. | 25 minutes | Present and briefly discuss content | -Discuss 4 Themes - 4 slides  -Open discussion about the content and how to deliver it to make it inclusive & engaging.   - Language/Appropriate tone? - Accessibility - Motivating? - Features?   -Write down any ideas on the Miro board between the two web browser frames  -Any features missing that you would add? |
| Aim: Sketch ideas for low-fidelity prototype.  Purpose: A better informed low-fidelity prototype and co-designer engagement. | 45 minutes | Whiteboard workshop | -**In smaller groups:**  -show content cards  Based on the larger group conversation and the content cards:  Have survivor scientists whiteboard ideas for display and delivery of content with us using Miro.  Keep in mind inclusivity and engagement when thinking about features/layouts.   - Sketch out ideas/examples based on content cards. - Go over organization/layout of the app. (animated videos, audio, text) - Gamification features? Would they be helpful? - What data should the app gather/track (ex. Goals, quiz scores)? - Draw low fidelity wireframe - Additional features/aspects that would make this app easier to use? |
|  | 15 minutes | Present whiteboard | **Back to bigger group:**  -Group presentation   - Show your wireframe and content specific ideas - Why were certain features/layouts used? - How would this layout help with inclusivity and engagement? |
|  | 20 minutes | Rank/Discuss | **Rank Features/Layout**  **Slide.do:**  -Based on what we’ve shown you  -Ranking  **Discuss rankings**  -Is there anything other delivery/engagement methods you can think of that would help cancer survivors with disabilities? |
| Aim: Close off and thank participants.  Purpose: Show appreciation for their time and better inform future co-design workshops. | 5 minutes | Closing Remarks | - Next Steps: Next Large Meeting - Discuss individual contributions in smaller working groups - email to come to reply to. - Thank the participants |
