# Supplementary material for: Developing an mHealth App for Empowering Cancer Survivors With Disabilities: Co-design Study
Source: JMIR Form Res. 2022 Jul 26;6(7):e37706. doi: 10.2196/37706 (PMC9364172; doi:10.2196/37706)
Supplement: Multimedia Appendix 3 [file formative_v6i7e37706_app3.docx]

**A Guide for Workshop #3 Prototype Development**

| **Aim *(what)* and Purpose *(why)*** | **Time Needed** | **Activity** | **Practicalities and Instructions** |
| --- | --- | --- | --- |
| Aim: To familiarize participants with activities and each other | 5 minutes | Introduction | -Discuss agenda for the day   - Intro - Breakout Session - Recap Breakout Rooms - Forum vs Feed (Tagging, etc.) - Closing Remarks |
|  | 15 minutes | Brief refresher of project purpose and aim | *-INTRO*   - *Educational Course* |
| Aim: Make modifications to the low fidelity prototype.  Purpose: A better informed high fidelity prototype and co-designer engagement. | 60 minutes | Breakout Session | -Show survivor scientists Wireframe  -Discuss good aspects VS any areas for improvement.   - Take notes on Miro board - Write down survivor scientists’ modifications in the copy of the Wireframe using Sticky Notes |
|  | 30 minutes | Modification Presentations | **Back to bigger group:**  - Discussion about modification.   - Explanation of modification. - Why does this modification make the interface better?   - Discuss Layout & Content |
|  | 35 minutes | Forum vs Feed (Tagging) | Discuss:   - Forum vs. Feed - *Perhaps show other forums and feeds* - Whether goals/comments should get added to it - Tagging terminology in forum |
| Aim: Close off and thank participants.  Purpose: Show appreciation for their time. | 5 minutes | Closing Remarks | - Open up for any questions - Thank the participants |
